# Empowering the younger generation increases their willingness for intergenerational reconciliation in the context of climate change

**DOI:** 10.1038/s41598-024-68145-9

**Published:** 2024-08-01

**Authors:** Janine Stollberg, Danja Bogdan, Eva Jonas

**Affiliations:** Department of Psychology, University of Salzburg, Hellbrunner Str. 36, 5020 Salzburg, Austria

**Keywords:** Human behaviour, Psychology and behaviour

## Abstract

Climate change can evoke intergenerational conflict. Structural inequalities and their unequal impact on generations can increase perceptions of collective victimhood among the younger generation (< 30 years) and bear the risk of social tensions between the young and the elderly. An experimental study (N = 434) showed that younger people perceived an increased risk of future victimhood. In line with a needs-based approach, the young reported an increased desire to pursue agentic intergroup goals, indicating a heightened need for agency. However, when the young received empowering messages that affirmed their ingroup agency, their willingness to reconcile with the old generation increased, whereas informing them about non-agentic ingroup behavior did not affect reconciliation (between-subjects manipulation). While empowering messages from the outgroup (“Grannies for Future”) that directly affirmed the young generations’ agency for climate change mitigation as well as empowering messages from the ingroup that indirectly affirmed ingroup agency in domains unrelated to climate change both addressed the need for agency, only outgroup empowerment promoted intergenerational reconciliation. However, empowerment did not affect support for collective climate action. We discuss empowerment as an avenue for resolving intergroup conflict in the context of climate change and possible consequences for climate action and social change.

“We have the choice: collective action or collective suicide” (António Guterres, 2023)

In his forceful speech on May 2nd, 2023, the ninth Secretary-General of the United Nations António Guterres drew attention to the fact that climate change not only calls for action but also for cooperation and solidarity^1^. This includes joint efforts of different nations, political parties, activist groups, and the broader population. However, structural inequalities between different societal groups can hamper joint efforts, when they lead to intergroup conflict. In the context of the climate crisis, an intergroup conflict can arise through perceived disadvantages, because some groups are more vulnerable but have less influence, while others are more responsible but do not take action to mitigate climate change^2,3^. Structural inequalities and resulting injustices between groups can foster intergroup conflict that may result in societal tensions, feelings of hurt, and resentments between groups that are difficult to repair^4^. A call to act is therefore always a call to reconcile within society, to create common ground for the former to thrive.

In the context of the climate crisis, conflicts or tensions between generations play a pivotal role. This is evident in social movements initiated by the young people (e.g., “Fridays for Future”), which emphasize the responsibility of the “last generation”. It becomes clear, as young people will suffer more from the consequences of climate change than older people^5^. Together, this highlights the intergenerational injustice of climate change and its consequences for the younger^6^. For instance, children under 10 will experience four times as many weather extremes, such as heat waves, as people over 60^5^. In addition, in developed countries the elderly (60 +) have the highest per capita carbon footprint due to household consumption behavior^7^, are less engaged in climate change mitigation^8^, and their lifestyle changes may oppose collective mitigation efforts^9^. This can be aggravated by the demographic development, predicting an increase in the elderly from almost 19% in 2022 to 27% in 2050 for Europe and North America^10^. Thus, people who are under 30 years old today, will heavily suffer from the consequences of climate change caused by the collective inaction and inertia of previous generations, which can create a sense of intergenerational injustice^6,11^. The problem is exacerbated by the fact that younger generations also have less impact on political decision processes than the elderly. For instance, during the last federal election in Germany in 2021, people under 30 years represented only 14.2% of those eligible to vote, whereas people over 60 years represented 38.8%^12^. To sum up, while solidarity is indispensable in the climate crisis, the intergenerational situation can lead to tensions and intergroup conflicts between younger and older people, potentially undermining cooperation and collective efforts that are necessary to mitigate climate change.

## The young generation as a victim of climate change

Due to this intergenerational injustice, younger people may perceive their generation as victims of climate change, as those who will suffer the most, and the older generation as perpetrators, as those who did not do enough to avoid the suffering. As a result, climate change may lead to perceptions of collective victimhood among the younger, which are not necessarily caused by active harm-doing, but by passive omission and denial of responsibility by the elderly. This may result in structural victimization of the younger generation. In contrast to physical victimization, structural victimization does not involve overt aggression or physical attacks. It is caused by social inequalities, different status positions and the unequal distribution of resources between groups that are maintained and normalized by the economic, political, or cultural system^3,13,14^. Aspects of structural victimization cumulate in the climate crisis and affect especially the younger^3^. For instance, young people will suffer from global warming first and foremost, but they are not yet in positions or institutions to influence political decisions on climate mitigation policies. These asymmetries between generations lead to structural victimization, which, like physical violence, can cause perceptions of collective victimhood. Collective victimhood is defined as “the psychological experience and consequences of (…) harm [by one group to another]”^15^. Therefore, in the context of the climate crisis, people of the younger generation may experience a sense of collective victimhood that makes them feel disregarded and unfairly treated by the elderly.

## Collective victimhood and the need for ingroup agency and control

When structural victimization is present for the younger generation in the context of the climate crisis, they should report a higher need for ingroup agency, the central need of victimized groups^16–18^. Perceptions of collective victimhood are often associated with need deprivation^15^. This is because structural victimization leads to unequal life opportunities that deprive basic human needs of the victim group. In particular, the need for agency, has been found to be deprived in group members that suffered from violent or structural forms of victimization^16–18^. Across different contexts of victimization (i.e., different cultural and ethnic contexts, aggressive and structural victimization) victimized groups reported a higher need to pursue agentic intergroup goals than communal goals during intergroup contact^19^. That is, victimized group members wanted to be perceived as confident and assertive (and not as understanding) when interacting with the perpetrator outgroup. This supports the need for agency as a central motive of victim groups in the context of structural victimization. We propose a similar pattern for the younger generation as structural victims of the climate crisis. As the young generation has less influence on political decision-making processes than the older generation, this should increase their desire to pursue agentic over communal intergroup goals when working toward collective climate change mitigation.

In addition, the younger generation's increased need for agency should also be reflected in an increased need for individual control. Agency is a central component of control definitions^20,21^. As a fundamental human need, the sense of control and agency can be experienced on the personal and the social level of the self^22,23^. That is, when agency is experienced as a group member, it can increase perceptions of individual agency and control^24^. While we expected intergenerational climate injustice to affect the need for agency primarily on the group level, we also expected it to increase the need for individual control over the situation. Therefore, we assumed that salient structural victimization in the context of climate change increases the younger’s need for ingroup agency and individual control.

## The role of ingroup agency for intergroup reconciliation and cooperation

Perceptions of collective victimhood among the younger generation should not only increase the younger’s need for collective agency, but at the same time affect the intergenerational relationship. Research on intergroup conflicts in the political domain has shown that people who perceived themselves as a part of a victim group, experienced less control and agency, and thus were less willing to improve the intergroup relation^25,26^. A large body of evidence has shown that the victim status made people less prosocial and less willing to reconcile with the perpetrator group^18^. We expect the same for the younger generation in response to the older generation. Young people under 30 years, who have a high need for ingroup agency due to their perceived victim status, should be less willing to reconcile with the elderly, as long as their need for agency has not been satisfied. In consequence, the younger’s willingness to engage in intergenerational reconciliation may only be achieved through specific need satisfaction, that is, by restoring their perception of ingroup agency. This can be done if young victims receive an empowering message from old perpetrators.

In addition to reconciliation, addressing the younger generation’s need for agency through empowering messages may also increase their willingness to cooperate with the older generation. It has been suggested that satisfying the need for agency of the structural disadvantaged group of young people can contribute to their willingness to cooperate with the advantaged group toward social change^27^. Therefore, we expected similar effects of empowerment in the context of climate change on the younger generation’s willingness to cooperate with the elderly toward climate change mitigation, as on their willingness to reconcile.

### Promoting reconciliation through empowering messages

Victim groups can be empowered by perpetrator groups: When victim group members received empowering (but not accepting) messages from the perpetrator group, they were more willing to reconcile with the perpetrator outgroup^18,28^. Empowering messages recognize the victim status and acknowledge the injustice, which can increase perceptions of control in the victimized group^29^. In addition, perpetrator groups holding a high-power position can empower victim groups by giving them voice, respecting their opinion and perspective, and acknowledging their competencies and achievements^30^. For instance, Israeli Jews who listened to an empowering speech by a German outgroup representative admitting Israeli Jews their strength and power to determine their own fate, reported a higher readiness to reconcile with the Germans than when they listened to an acceptance message that emphasized understanding and their ingroup’s suffering^28^. Thus, empowering outgroup messages can restore a sense of ingroup agency in victimized group members, which then helps to improve intergroup relations. For the context of the climate crisis, we assume that young people who receive an empowering message from representatives of the older generation that acknowledges the younger’s competence and influence in mitigating climate change (e.g., social movement efforts), admits the intergenerational injustice, and gives them a voice will increase their willingness to reconcile with the elderly more than those who did not receive such an empowering message.

### Empowerment through the affirmation of ingroup agency

While empowerment through an outgroup is often desirable, it is not always feasible. In the face of the climate crisis, interactions between societal groups may become even more difficult, as threat perceptions can increase ethnocentric responses and promote the social divide^31,32^. Furthermore, (future) social inequalities affecting the (younger) minority may not be recognized by the vast (older) majority. Here, another possibility to restore a sense of agency in the younger can be the affirmation of agency at the group level, as feelings of control and influence can be experienced and demonstrated as an individual and as a group member^22,23,33^. The affirmation of ingroup agency should be independent of the context in which one perceives one’s ingroup as an “autonomous agent who is capable to achieve its goals” (definition of group agency^34^).That is, the affirmation of agency can also occur in an indirect fashion when the need for agency is threatened in a different context^35^.

First evidence for the successful indirect restoration of ingroup agency stems from intergroup conflict research: Israeli Jews who read a text affirming the competence and strength of their nation in several domains, were more willing to donate to Palestinian humanitarian aid agencies than those who affirmed Israeli morality or who did not engage in ingroup affirmation^26^. This is in line with our assumption that the restoration of ingroup agency is the key motivational process driving intergroup reconciliation in the context of an intergenerational conflict in the climate crisis. We propose that the affirmation of collective agency can occur in a direct fashion (i.e., through outgroup messages supporting the younger’s competence and influence on climate mitigation efforts) or in an indirect fashion (i.e., through ingroup messages supporting the younger’s competence and influence independent of the climate crisis). We assumed that both, outgroup and ingroup empowerment should be effective in restoring a sense of ingroup agency in the younger generation, which then increases the younger’s willingness to reconcile with the elderly in the climate crisis. We tested this prediction in an experimental study, thereby for the first time, directly comparing outgroup and ingroup empowerment effects on the victimized group’s willingness to reconcile.

### Need for collective agency and collective climate action

Restoring the need for agency of the victimized group may not only affect reconciliation willingness but promote collective climate action support. Perceptions of ingroup agency are a central determinant of support for collective action in general^36^, and for collective climate action in particular^24,37,38^. Recent meta-analytic findings have also shown that empowering the victim group during intergroup contact increased their support for social change^39^. Thus, providing the younger generation with a sense of ingroup agency through empowering ingroup or outgroup messages should not only increase conciliatory tendencies toward the elderly, but may also positively affect their support for collective climate change mitigation. Therefore, we assessed support for climate change mitigation together with conciliatory tendencies as dependent variables.

## The present study

We used an experimental approach to investigate climate change as an intergenerational conflict and the impact of structural victimization and empowerment on young people’s willingness for intergenerational reconciliation and support for collective climate action. Therefore, we independently manipulated the salience of structural victimization of the young generation (i.e., salient vs. not salient) and empowerment as between subject variables. In particular, we tested whether empowering messages from the older generation outgroup (“Grannies for Future”) vs. empowering messages from the younger generation ingroup (“agency affirmation”) increased the younger generation’s willingness to cooperate and reconcile with the older generation, by providing the younger with a sense of ingroup agency. We compared this to a non-empowering control condition (i.e., making non-agentic ingroup behavior salient). We hypothesized that young people under 30 years report a higher need for individual control and ingroup agency (than for communion), if they perceive themselves as victims of climate change (H1 & H2). Then, empowering the victimized group (“the younger generation”) through messages from the perpetrator group outgroup (“the older generation”) that recognize the situation and acknowledge the power and influence of the younger should increase the younger’s willingness to cooperate and reconcile with the elderly; empowering messages from the victimized ingroup that affirm the ingroups’ agency in other contexts than climate change should have the same effect, whereas non-empowering information about typical but non-agentic ingroup leisure time activities should not (H3). Further, we assumed empowering messages to have positive effects on support for collective climate action (H4). All hypotheses, the experimental design, and analyses were pre-registered on open science framework, https://osf.io/jr75w/?view_only=d9a2c51935834883ba2c86a04978314b.

## Method

### Participants and design

The study was conducted as an online experiment on “Opinions regarding current societal developments” with random assignment to conditions. The design was 2 Structural Victimization (salient/ not salient) × 3 Empowerment (outgroup/ingroup/ non-empowering) between subjects. Participants were recruited via social media channels, online recruitment platforms, and through advertisement on public places.

### Sample

We determined our sample size by an a priori power analysis using G* Power^40^. To detect the predicted interaction of salient victimization by empowerment on reconciliation willingness, with an effect size of *d* = 0.30, statistical power of 80%, and an error probability of 5%, we needed an estimated sample size of *N* = 432. We based our estimation of effect size on empowerment effects on reconciliation for victimized groups obtained in previous studies^28^. We recruited a slightly larger number of *N* = 451 participants from German speaking European countries. To ensure that all participants belonged to the ingroup of the younger generation, we excluded 19 participants who were older than 30 years, resulting in a final sample of *N* = 434. The participants were *M* = 24.62 (*SD* = 2.94) years old, 274 identified themselves as female, 158 as male, and two as divers.

### Procedure

After giving informed consent, participants filled in demographic questions before they received information about the structural victimization of the younger generation in the climate crisis (vs. neutral facts about the Earth). To assure that participants read the information attentively, they had to answer two questions about the content. This was followed by two items measuring perceived victimhood in the victimization salient condition. In the neutral control condition, participants responded to these items at the end of the experiment to avoid victimization salience. Then, participants’ need for control, ingroup agency, and ingroup communion were assessed before they were randomly assigned to one of three empowerment conditions. After reading the (non-) empowering message, participants reported their willingness to reconcile with the older generation, expressed their support for cooperation and for collective climate mitigation strategies. At the end of the questionnaire, participants had the opportunity to express their opinion on whether they perceive an intergenerational conflict in the climate crisis and to participate in a raffle. Then, they were fully debriefed and thanked for their participation. Eight vouchers about 50€ were raffled and handed out after data curation was completed.

The experiment complied with APA 7th ethical guidelines and the procedure was approved by the University of Salzburg Ethics Board. The study was conducted according to these guidelines, and informed consent was obtained from all participants.

### Manipulation of structural victimization salience

We manipulated structural victimization salience between participants. We made the structural victimization of the younger generation salient to half of the participants and compared this to neutral facts about the Earth (e.g., temperature, number of Mediterranean seas), which has been widely used as a neutral control condition in research on motivated reactions to climate change^24,32^. Participants in the victimization salient condition received information about “the intergenerational conflict in the climate crisis”, including survey results and bar graphs, showing that the younger generation will suffer more from climate change than the elderly and has done more to reduce its carbon footprint, whereas the older generation has done less. Moreover, it was made salient that the number of eligible voters is twice as high in the older generation, indicating their greater political power. In both experimental conditions the information was presented as a bogus newspaper article, based on real statistics and facts.

#### Perceptions of collective victimhood

We asked participants with two items, whether they felt unjustly treated and disregarded by the older generation, *r(432)* = 0.75, *p* < 0.001, to assess their perceptions of collective victimhood. This served as a manipulation check for the victimization salience manipulation. Participants in the victimization salience condition reported similar levels of collective victimhood, *M* = 4.35, *SD* = 1.42, as participants in the not salient condition, *M* = 4.11, *SD* = 1.52, *t*(432) = 1.69, *p* = 0.092, *d* = 0.16. However, when structural victimization was salient, participants’ sense of collective victimhood was above the scale mid-point, *t*(211) = 3.56, *p* < 0.001, *d* = 0.25, whereas in the no salient condition it was not, *t*(221) = 1.06, *p* = 0.290, *d* = 0.07. Looking at the two aspects of collective victimhood (i.e., “feeling disregarded by the elderly” and “feeling unjustly treated by the elderly”) separately, revealed that victimization salience increased the young participants perceptions of feeling disregarded (*M* = 4.40, *SD* = 1.48 vs. *M* = 4.08, *SD* = 1.56), t(432) = 2.19, *p* = 0.029, *d* = 0.21, but not of feeling unjustly treated by the elderly (*M* = 4.30, *SD* = 1.61 vs. *M* = 4.14, *SD* = 1.65), *t*(432) = 1.01, *p* = 0.315, *d* = 0.10. In addition, we asked participants in an open question at the end of the questionnaire about their opinion, whether they perceived an intergenerational conflict in the context of climate change. The results of the qualitative answers are available in the supplements (see S Table 2). They show that 67% perceived some kind of intergenerational conflict, whereas 16% did not (17% were not categorizable). Thus, perceptions of collective victimhood in the context of climate change are probably an issue for the younger generation, independent of structural victimization salience.

### Need for ingroup agency, communion, and individual control

After reading about intergenerational structural inequalities (or facts about the Earth), participants rated their need for ingroup agency and communion. Therefore, we initially selected 20 items from the Circumplex Scales of Intergroup Goals (CSIG)^41^ that best fit into the context of structural victimization due to climate change. The CSIG has been shown to reliably capture the need for agency and the need for communion at the intergroup level across different victimization contexts^19^. We constructed the scales to measure the need for ingroup agency and the need for ingroup communion on the basis of an exploratory factor analysis (The full analysis is available in the Supplementary Material (see Supplementary Fig. 1 and S Table 1)). The younger’s need to pursue agentic intergroup goals was measured with 8 items from the high agentic dimensions “be respected” and “be assertive” that built the first factor, and the younger’s need to pursue communal intergroup goals was measured with 6 items from the high communion dimensions “be understanding” and “be cooperative”, that built the second factor. Participants were asked to indicate their agreement on a 7-point scale (1 = *disagree at all* to 7 = *fully agree*): *“I think, for us as young generation it is important that…”, e.g., “…we are assertive”, “…the older generation respects what we have to say”,* for agentic intergroup goals, α = 0.87, and, e.g., *“…we understand their point of view”, “…we appreciate what they have to offer”,* for communal intergroup goals, α = 0.83.

In addition, we assessed participants individual need for control with four items from Shnabel and Nadler^16^ adapted to the context of climate change, e.g., *“I would like to have more influence on climate change”, “I would like to have more control over the cooperation with the older generation in fighting climate change*”, α = 0.76.

### Manipulation of empowerment

Participants randomly received one of three empowerment texts (outgroup/ ingroup/ non-empowering). In the outgroup empowerment condition, they were provided with a bogus speech based on real newspaper articles by a “Grannies for Future” activist entitled “We have to learn from each other”. The speech contained the following empowering elements^18^: Recognition of injustice, interest in the younger generation’s opinion and giving them voice, offering reparation, and acknowledgment of the younger generation’s achievements and capabilities in fighting climate change. In the ingroup empowerment condition, they received a text on “Young world changers”, that empowered the younger generation with examples of young activists who have achieved important societal goals in different areas, such as education, equal rights, and peace. In the third condition, participants received a non-empowering text that also made the younger generation’s ingroup salient but without emphasizing the group’s agency, by describing typical leisure time activities of the younger, such as meeting friends, relaxing, or cooking.

After reading the messages, participants were asked whether they felt that they, as part of the younger generation, could actively change things, and whether they thought that they could influence social interactions. Both items were combined and served as a manipulation check for empowerment, *r(432)* = 0.76, *p* < 0.001. As participants in both empowerment conditions felt more empowered (outgroup: *M* = 5.39, *SD* = 1.10, ingroup:* M* = 5.60, *SD* = 1.17) than participants in the non-empowering condition (*M* = 4.92, *SD* = 1.17), 95% CI for mean differences outgroup-non-empowering condition [0.22, 0.74], ingroup-non-empowering condition [0.42, 0.96], outgroup-ingroup [− 0.48, 0.06], *F*(2,432) = 13,46, *p* < 0.001, η = 0.06, we deemed our empowerment manipulation successful.

### Dependent variables

All variables were measured on a 7-point scale (1 = *not at all* to 7 = *very much*). After item analyses, mean scores for each variable were computed.

#### Willingness to reconcile

We measured willingness to reconcile with 10 items adapted from Shnabel and colleagues (2009).^28^ Participants indicated whether the previous information, e.g., “…*increases my willingness to work for a better relationship between the generations., “… creates a better image of the older generation in my eyes.”, “… makes me more optimistic about the future relations between my generation and the older generation.”,* α = 0.95.

#### Willingness to cooperate with the elderly outgroup

Willingness to cooperate with the older generation in pursuing the common goal of mitigating climate change was measured with five self-generated items, e.g., “*I would like to continue working with the older generation in the fight against the consequences of climate change.”*, α = 0.72.

#### Support for collective climate action

We measured participants’ intentions to support six different collective climate change mitigation behaviors that were created ad-hoc, e.g., “*I am willing to participate in demonstrations for the most comprehensive, rapid, and efficient climate protection measures possible.”, ·”I am in favour of a carbon dioxide tax for private individuals, trade, and industry.”, “I am willing to sign (online) petitions for climate protection”,* α = 0.88.

## Results

### Victimization effects on individual need for control

We conducted an independent sample t-test to test whether salient structural victimization increased participants’ need for individual control (H1). The younger reported equal levels of need for individual control, when structural victimization was salient, *M* = 4.90, *SD* = 1.14, compared to the when it was not, *M* = 5.08, *SD* = 0.98, *t*(432) = − 1.71, *p* = 0.087, *d* = − 0.17. Salient structural victimization in the context of climate change did not change individual perception of control (Table 1).

**Table 1:** Descriptive values for need perceptions depending on victimization salience.

			Collective needs		Individual need
			Agency goals	Communion goals	Need for control
Structural Victimization	Salient	*M (SD)*	5.65 (0.89)	5.43 (0.93)	4.90 (1.14)
	Not Salient	*M (SD)*	5.73 (0.81)	5.43 (0.88)	5.08 (0.98)

Means and standard deviations for measures of collective and individual needs as a function of experimental condition (structural victimization by climate change salient vs. not salient).

### Victimization effects on need for ingroup agency and communion

We assumed structural victimization of the younger generation to increase the younger’s need for ingroup agency but not their need for ingroup communion (H2). To test this hypothesis, we conducted a repeated measures ANOVA with structural victimization salience as between-subjects factor and the need for agency and the need for communion as within-subjects factor. The results show that structural victimization salience did not affect participants need for ingroup agency or communion, main effect, *F*(1,432) = 0.35, *p* = 0.554, η ^2^ = 0.001, within-between interaction. *F*(1,432) = 0.94, *p* = 0.333, η^2^ = 0.002. However, as indicated by the multivariate interaction, *F*(1,432) = 42.38, *p* < 0.001, η^2^ = 0.09, the younger were more motivated to pursue agentic goals than communal goals when working with the elderly toward climate change mitigation. This multivariate effect emerged in the structural victimization salient condition, *F*(1,432) = 28.63, *p* < 0.001, η^2^ = 0.06, 95% CI for mean difference [0.19, 0.41], as well as in the not salient condition, *F*(1,432) = 15.01, *p* < 0.001, η^2^ = 0.03 95% CI for mean difference [0.11, 0.33] (see Table 1), indicating that all young participants had a higher need for ingroup agency than for communion, independent of structural victimization salience.

### Exploratory analysis: correlations between needs and subjective perceptions of collective victimhood

To test whether a subjective sense of collective victimhood was associated with an increased need for agency and control independent of victimization salience, we looked at the correlations between perceived collective victimhood, need for ingroup agency, ingroup communion, and individual control (Table 2). The results were supportive of the need-based-model approach: Young people who perceived their ingroup more as victim of climate change reported an increased need for individual control, *r* = 0.43, *p* < 0.001, wanted to pursue agentic intergroup goals, *r* = 0.25, *p* < 0.001, but showed a decreased need to pursue communion goals, *r* = − 0.10, *p* < 0.042. Together, these results support the notion that perceptions of collective victimhood in the context of an intergenerational climate change conflict are associated with a higher need for ingroup agency and individual control.

**Table 2:** Correlations between perceived collective victimhood and needs.

	Variable		1	2	3
1	Perceived collective victimhood	*r*			
		*p*			
2	Need for ingroup agency	*r*	0.25		
		*p*	< 0.001		
3	Need for ingroup communion	*r*	− 0.10	0.57	
		*p*	0.042	< 0.001	
4	Need for individual control	*r*	0.43	0.51	0.15
		*p*	< 0.001	< 0.001	0.002

Correlations between participants subjective sense of collective victimhood of the young generation and their ingroup’s need for agency, communion, and individual control.

### Effects of empowerment on reconciliation and cooperation

**Figure 1:**
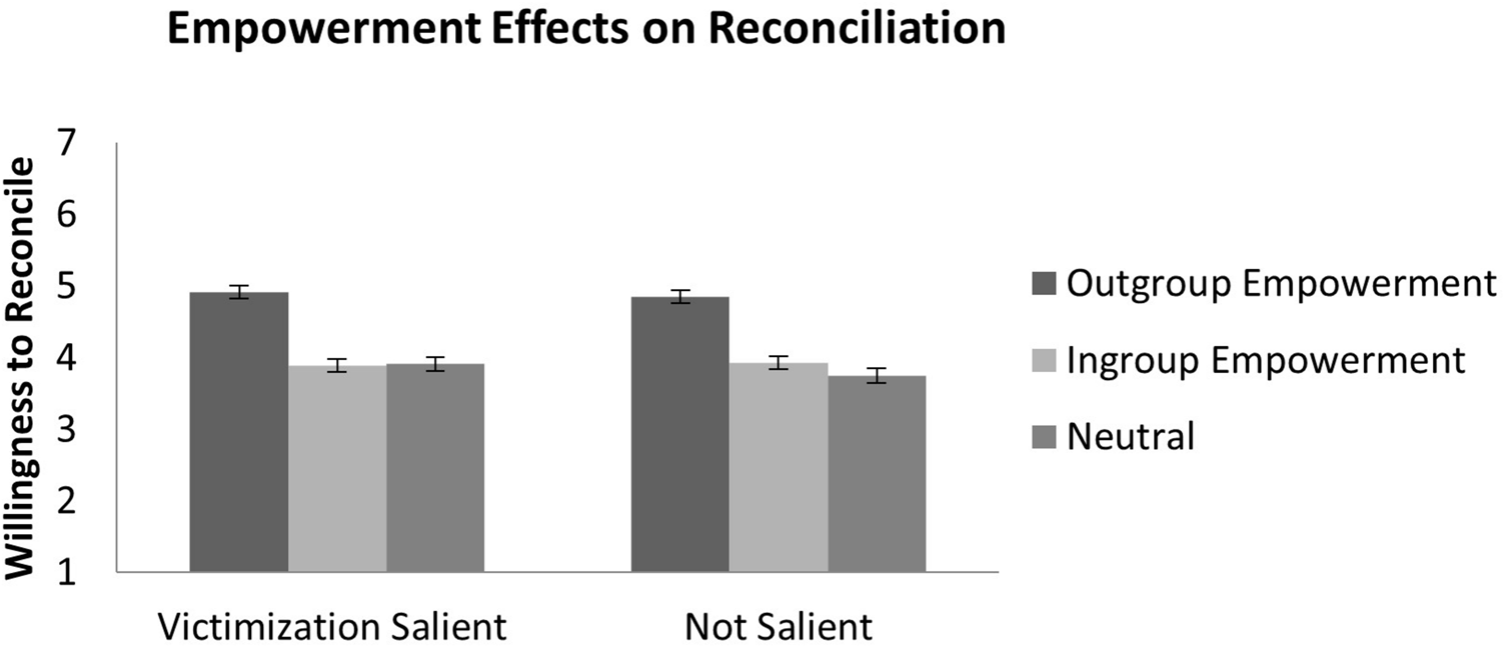
Direct effects of empowerment on intergenerational reconciliation. The figure shows the direct effect of empowerment on intergenerational reconciliation. This is the test of the main hypothesis. Empowerment by the outgroup (“Grannies for Future”) increased significantly, *p* > 0.001, the youngers’ willingness to reconcile with the elderly, independent of whether the structural victimization of the younger generation was salient. Error bars indicate standard errors of the mean for empowerment conditions.

To test our main hypothesis (H3) that empowering the younger generation increases their willingness to reconcile and cooperate with the older generation, we conducted a 2 Structural Victimization (salient/not salient) × 3 Empowerment (outgroup/ingroup/non-empowering) ANOVA on their willingness to reconcile and cooperate, respectively. The distribution of participants to conditions can be seen in Table 3. For reconciliation, the results showed a main effect of empowerment, *F*(2,428) = 40.48, *p* < 0.001, η^2^ = 0.16, (see Fig. 1). When the younger received an empowering message from the “Grannies for Future” representing the elderly outgroup, they reported more willingness to reconcile with the older generation compared to those who received non-empowering information about their ingroup’s leisure time activities, 95% CI for mean difference [0.80, 1.30], or to those who were provided with a message that affirmed their ingroup’s agency in other domains than climate change, 95% CI for mean difference [0.71, 1.23]. The effects of empowerment on reconciliation were independent of structural victimization salience, *F*(2,428) = 0.31, *p* = 0.735, η^2^ = 0.001. The main effect of structural victimization was also not significant, *F*(1,428) = 0.28, *p* = 0.595, η^2^ = 0.001. The same analysis for willingness to cooperate as dependent variable, did neither show the expected empowerment nor other effects. The results of the 2 Victimization (salient/not salient) × 3 Empowerment (outgroup/ingroup/non-empowering) ANOVA on cooperation revealed no significant effects, main effect victimization, *F*(1,427) = 1.59, *p* = 0.208, η^2^ = 0.004, main effect empowerment, *F*(2,427) = 0.42*, p* = 0.656, η^2^ = 0.002, interaction victimization x empowerment, *F*(2,427) = 0.65, *p* = 0.523, η^2^ = 0.003.

**Table 3:** Number of participants for each condition.

		Empowerment		No empowerment	
		Outgroup empowerment	Ingroup empowerment		
Structural victimization	Salient	n = 74	n = 68	n = 70	N = 212
	Not salient	n = 78	n = 63	n = 81	N = 222
		N = 152	N = 131	N = 151	N = 434

Distribution of participants to conditions.

### Effects of empowerment on support for climate change mitigation

We expected empowerment to increase the younger’s intentions to support collective climate change mitigation behavior (H4). To test the hypothesis, we ran a 2 Structural Victimization (salient/not salient) × 3 Empowerment (outgroup/ingroup/non-empowering) ANOVA on support for climate action. The results showed that empowerment did not affect the willingness of the younger to support collective climate action measures, main effect *F*(2,428) = 1.44, *p* = 0.237, η^2^ = 0.01, and empowerment x structural victimization interaction effect, *F*(2,428) = 1.41, *p* = 0.245, η^2^ = 0.01. However, and not expected, there was a main effect of structural victimization, *F*(1,428) = 3.99, *p* = 0.046, η^2^ = 0.01. Salient structural victimization of the younger generation decreased their intentions to support collective climate action measures (*M* = 4.24, *SD* = 1.53), compared to when it was not salient (*M* = 4.53, *SD* = 1.45).

### Exploratory analysis: need for ingroup agency explains increased reconciliation in victims of climate change

**Figure 2:**
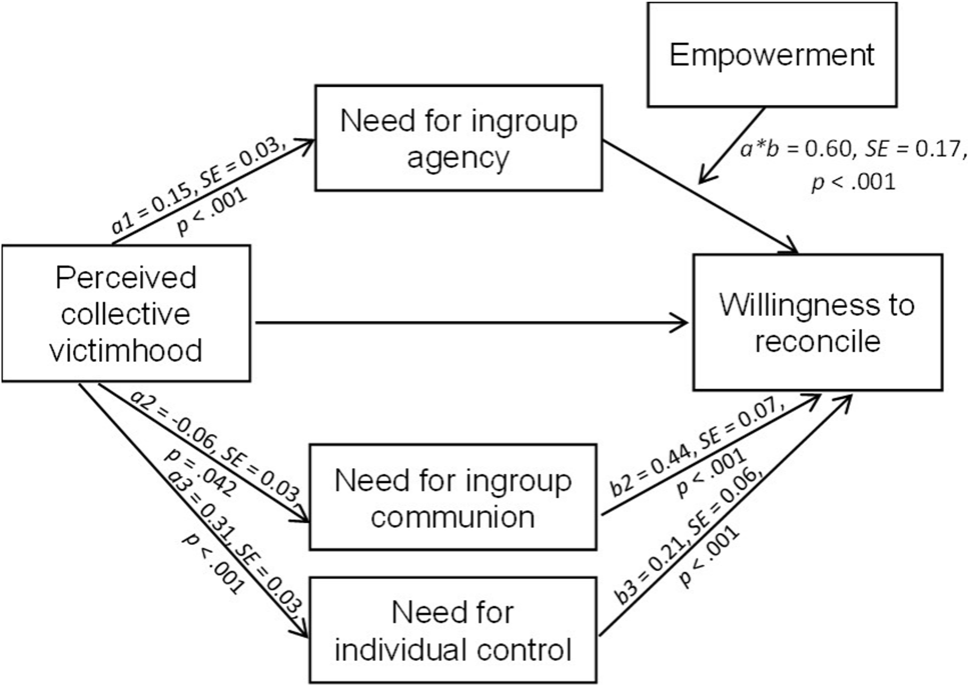
Need for ingroup agency as underlying process: empowerment buffers negative victimhood effects on reconciliation through an increased need for ingroup agency. The figure shows the results of the moderated mediation analysis, supporting the need for agency as underlying motivational process. Participants who perceived their ingroup of the young generation as victim of climate change reported less willingness to reconcile with the elderly IE 95% [− 0.16, − 0.05], unless they received an empowering message from the outgroup IE 95% [− 0.07, 0.03] or the ingroup IE 95% [− 0.04, 0.04].

In addition to our preregistered analyses, we conducted an exploratory moderated mediation analysis to examine the underlying needs-based process of empowerment on reconciliation for victims of climate change. We expected empowering messages from the ingroup and the outgroup to satisfy in particular, the heightened need for ingroup agency in those who perceived their young generation as a victim of climate change. Since the empowering messages emphasized the capability of the young generation’s ingroup to actively exert influence on important societal outcomes, this should address the need for agency as a group member. To test this assumption, we ran a moderated mediation analysis, using the process 4.3 macro for SPSS (Model 14)^42^, with collective victimhood as predictor, need for ingroup agency as mediator, willingness to reconcile as outcome variable, and empowerment as moderator (Helmert contrast coded) of the relation between needs and reconciliation (see Fig. 2). We used participants subjective sense of collective victimhood as continuous predictor instead of manipulated structural victimization, as perceptions of collective victimhood did not differ significantly between structural victimization salience conditions. Additionally, we tested for need for individual control and need for ingroup communion as underlying process variables, including them as alternative mediators into the model.

In line with a needs-based model approach, we found support for the moderated mediation for ingroup agency, as indicated by the significant interaction of empowerment by ingroup agency, *b* = 0.60, *t*(432) = 3.41, *p* < 0.001, but not for ingroup communion, *b* = − 0.05, *t*(432) = − 0.38, *p* = 0.700, or individual need for control, *b* = − 0.07, *t*(432) = − 0.62, *p* = 0.536. Perception of collective victimhood were associated with an enhanced motivation to pursue agentic intergroup goals, *b* = 0.15, *t*(432) = 5.39, *p* < 0.001, which in turn decreased the willingness to reconcile with the older generation when the younger’s need for ingroup agency was not addressed by empowering messages, IE 95% CI [− 0.16, − 0.05]. Ingroup empowerment as well as outgroup empowerment buffered the negative effect of ingroup agency on reconciliation, IE 95% CI [− 0.04, 0.04] for ingroup empowerment, and IE 95% CI [− 0.07, 0.03] for outgroup empowerment. This supports the need for ingroup agency, which is reflected in the desire to pursue agentic intergroup goals, to underlie victimhood effects on the youngers’ willingness to reconcile with the elderly. Importantly, independent of whether participants could restore a sense of group agency through empowering messages by the perpetrator outgroup or by affirmation of ingroup agency, both addressed the need for ingroup agency.

## Discussion

We investigated the climate crisis as an intergroup conflict between the younger generation (< 30) and the older generation (60 +) in an experimental study. Our results showed that structural inequalities (i.e., power asymmetries and responsibilities) between generations in the context of climate change elicited perceptions of collective victimhood in the majority of the young participants. Participants felt disregarded and unjustly treated by the older generation. Although perceptions of collective victimhood did not differ significantly between experimental conditions that made structural victimization (not) salient, they varied substantially within participants.

Participants who perceived high levels of collective victimhood also reported a strong need to pursue agentic intergroup goals but not communal goals. That is, young people who experienced their generation as victims of the climate crisis had a strong desire to be perceived as assertive and respected (but less as accepting and understanding) in interactions with the older generation. This is in line with our prediction that perceptions of collective victimhood are associated with an increased need for ingroup agency. Moreover, it supports the assumptions of the needs-based model of reconciliation for a victim group in an intergenerational conflict situation.

Addressing the young participants’ need for ingroup agency with an empowering message from the elderly outgroup increased their willingness to reconcile with the older generation, whereas ingroup empowerment did not. Outgroup and ingroup empowerment acknowledged the achievements and capabilities of the younger generation. While the outgroup message directly addressed the younger’s agency in the intergenerational conflict of climate change, the ingroup message indirectly affirmed the younger’s agency in other domains unrelated to climate change. Empowerment by the outgroup (but not by the ingroup) had a positive main effect on reconciliation, independent of perceived or manipulated victimhood. When an outgroup activist from “Grannies for Future” acknowledged the young generations achievements and gave them a voice, then the young participants’ reported a greater willingness to reconcile with the elderly. Unexpectedly, neither outgroup nor ingroup empowerment affected the youngers’ support for collective climate action.

The results of an exploratory conditional process analysis lend further support to the assumed needs-based process from perceived collective victimhood to reconciliation via the need for ingroup agency. Here, empowering messages from the outgroup and the ingroup buffered the negative effect of victimhood perceptions on reconciliation by addressing the need for ingroup agency. However, only outgroup empowerment increased the young generation’s willingness to reconcile with the elderly directly, whereas ingroup empowerment only buffered the intergenerational divide. This supports the need for agency as a central motive for intergenerational reconciliation among young people, but also speaks to the unique power of outgroup empowerment for reconciliation.

## Intergenerational reconciliation: a needs-based process

Our findings support the needs-based model of reconciliation^16^, the first time for an intergenerational conflict. We found that young people under 30 years who perceive their generation as victims of climate change report an increased need for ingroup agency. This extends previous findings that found victimized groups in different context of physical transgressions to experience a heightened need for ingroup agency^19^ by turning to structural victimization in the context of climate change. It is also in line with findings showing structural inequalities to cause victimization in intergroup situations: Member of a low status group who perceived their ingroup status as illegitimate also reported an increased need for agency^43^. Here, in the context of climate change, young people who felt unjustly treated by the elderly and who perceived their generation as a victims of climate change, also reported a higher need for ingroup agency than for ingroup communion. The need for agency was reflected in a higher motivation to pursue agentic intergroup goals (i.e., to be perceived as an assertive and respected interaction partner) in intergenerational interactions. This supports the need for agency as the basic need of victimized groups, and it is in line with research showing that different victim groups reported a higher need to pursue agentic intergroup goals than communal goals during intergroup conflicts^19,25,26^.

### Direct empowerment through the outgroup

When the need for agency was addressed through an empowering outgroup message, the younger were more willing to reconcile with the older generation to mitigate climate change. This highlights the positive effect of intergenerational communication in the perceived intergroup conflict of climate change. When old people recognize what the older generations have failed to do and acknowledge what the younger generation has already achieved, it seems to be a powerful tool for intergenerational reconciliation. This result is in line with the basic findings of the needs-based model of reconciliation in different political and historical intergroup conflicts^18,28^, and supports previous findings showing the positive effect of low-status group empowerment on reconciliation^30^. Messages of the perpetrator group might be even more effective to promote intergroup reconciliation in the context of climate change than empowerment by ingroup agency, as they may not only satisfy the need for agency but also build a sense of trust^44^. If the acknowledgment of the young generation’s capabilities and achievements is expressed by a member of the old generation, the younger will be more ready to approach the elderly and see their good intentions. It is possible that the “Grannies for Future” are particularly effective as senders of the message because they are perceived as a credible source, as those who honestly care about climate change and engage in reparation.

### Indirect empowerment through the affirmation of ingroup agency

In addition to outgroup empowerment, we included an ingroup empowerment condition, in which empowerment could be achieved indirectly through the affirmation of successful achievements of the younger generation in domains unrelated to the climate crisis. While previous studies found positive effects of personal agency affirmation on reconciliation^25^, we could not replicate these effects for ingroup agency affirmation in the ingroup empowerment condition. Although we found support for both ingroup empowerment and outgroup empowerment to address the need for ingroup agency and to buffer the negative effects of perceived victimhood on reconciliatory tendencies, only outgroup empowerment further increased the younger generation’s willingness to reconcile with the elderly.

Nevertheless, our findings emphasize that addressing the victim group’s need for agency is more important for ameliorating intergroup conflict than the mere affirmation of group membership (i.e., thinking about typical but non-agentic group behavior). This adds to findings showing that the affirmation of ingroup agency compared to non-affirmation or the affirmation of different needs increased constructive and prosocial behavior towards outgroups^26^. It further supports the notion that agentic ingroups in particular are attractive to people with high needs for agency and control^45^, and that the affirmation of ingroup agency can restore a deprived need for agency in another domain^35^. This process is better explained by social identity-based processes^26,34^ than by mere self-affirmation^46^.

## Empowerment increases reconciliation but not cooperation towards climate action

Although empowerment had positive effects on reconciliation, we did not observe an effect on the younger’s willingness to cooperate with the elderly for joint action against climate change. While cooperation towards a common goal can be a consequence of reconciliation and is desirable for social change^27^, the specific context of climate change may explain why we did not find any effects on cooperation. It is possible that cooperation with the older generation was perceived by the younger as a threat to their group’s autonomy for sustainable action. The need for collective autonomy has been discussed as another need beyond agency and acceptance that can be thwarted in the context of victimization, especially in unequal power relations^47^. Perhaps, the younger have the feeling to compromise and cannot force their idea of a sustainable lifestyle and their favourite strategies to mitigate climate change, which threatens their need for autonomy and prevents them from cooperation. Future studies should consider the need for autonomy as an independent and relevant need in the context of inter(generational)group conflicts.

## Need for ingroup agency and collective action

Contrary to our expectations, we did not observe empowerment effects on support for climate change mitigation behavior. Moreover, we found that making victimization salient slightly decreased the younger’s willingness to support collective climate actions compared to participants in the control condition. Although not expected, this finding is in line with other studies reporting difficulties to increase collective efficacy perceptions through experimental manipulations in the context of climate change^47,48^. Rational perceptions of ingroup agency and motivational tendencies to want high group agency may interfere when the negative consequences of climate change are salient^24,48^. Other factors, such as supportive ingroup norms, may be more predictive of collective climate action support^37^. However, results from a large-scale multinational study support the notion that empowerment increases victims support for social change across different ethnic and gender contexts^39^. Future studies should rule out whether this finding replicates in the context of climate change intergroup conflicts using different measures.

## Perceptions of collective victimhood in the context of climate change

### Structural victimization and temporal scope

Our study provides empirical support that structural victimization in the context of climate change affects intergenerational conflict perceptions. In addition to other structural inequalities that emerge between the global North and the global South^2,3^, the climate crisis fuels collective victimhood perceptions between generations within national societies. This form of structural victimization by the climate crisis deserves more attention, as its consequences for group victim beliefs and intergroup outcomes are barely understood. Research on collective victimhood has shown that different perceptions of collective victimhood are associated with different intergroup outcomes, such as ethnocentrism and conspiracies^49^, distrust and conflict exacerbation^50^, negative attitudes toward outgroup members^51,52^, but also prosociality toward other victim groups^52,53^. The construction of intergenerational victim beliefs in the context of climate change may differ from collective victimhood perceptions in other contexts, as they are less about active harm-doing but more about passive omission. Moreover, climate change victimhood is an anticipated form of victimhood, as the consequences lie in the future. Research on historical victimization (i.e., “siege mentality”^50^) has shown that collective victim beliefs persist from past group victimization into the present and affect cognition and motivation of group members. The same may be true for anticipated climate change victimhood that is experienced in the future but affects group cognition and intergroup relations in the present.

## Limitations

### Manipulation of structural victimization

A limitation of this study is that we were not able to successfully manipulate structural intergenerational victimization in the context of climate change. We did not find any difference in collective victimhood beliefs for participants who were provided with information about structural victimization of the younger generation comparted to participants that received no such information. However, victimhood beliefs varied substantially among participants with the majority perceiving some type of intergenerational conflict. It is possible that climate change victimhood beliefs of the young generation are already central to the younger’s ingroup identification^54^, or that individual victim beliefs are more important in this particular context.

### Different effects of ingroup and outgroup empowerment

Another limitation is the lack of empowerment effects on reconciliation for the ingroup message. This may be due to differences between the outgroup and ingroup empowerment manipulations. In both conditions, participants read about the competencies and achievements of the younger generation, a central component of empowerment^30^. However, the outgroup message was a motivational speech that included further aspects of empowerment, such as recognizing the injustice and the victim status of the younger^29^, thereby reemphasizing the intergroup conflict. Thus, the outgroup message may have been better suited to empower the younger in the intergenerational conflict of climate change.

The positive effects of outgroup empowerment on conciliatory tendencies, may also be traced back to the speaker from “Grannies for Future” as representative of the outgroup generation over 60. Membership in the perpetrator group was determined by age. However, and in line with a social identity perspective, there may be subgroups within the old generation, such as the Grannies for Future that support climate change mitigation, and others that do not care, or that are explicitly against climate change mitigation measures. Some of these perpetrator subgroups may be better suited to empower the younger because they are more trustworthy as supporters of climate action than others who oppose it. Trust has been shown to be a secondary route to reconciliation in addition to agency in victimized groups^44^. Nevertheless, as all subgroups share a common identity (i.e., the old generation), they should be equally effective in sending empowering messages to increase the younger’s willingness to engage in intergenerational reconciliation^55^. Future studies should examine the potential and underlying processes of different generational subgroups for reconciliation.

## Practical implications

This study provides first evidence that acknowledging the capabilities of the younger generation and giving them a voice can help ameliorate the intergenerational conflict situation in the context of climate change. Our findings support the role of empowerment in addressing the younger generation’s need for agency as a central aspect of intergenerational communication, while denying them skills and voice and reassuring them that the elders are in control of the situation could make the situation worse by further undermining their need for agency.

## Supplementary Information

Supplementary Information.

## Data Availability

All stimulus material and anonymized study data are publicly available through the Open Science Framework, OSF. https://osf.io/x38zc/?view_only=1ea7fac02aa54d5487e7520fe3982a82.
