# Author Correction: Empowering the younger generation increases their willingness for intergenerational reconciliation in the context of climate change

**DOI:** 10.1038/s41598-024-74254-2

**Published:** 2024-10-10

**Authors:** Janine Stollberg, Danja Bogdan, Eva Jonas

**Affiliations:** Department of Psychology, University of Salzburg, Hellbrunner Str. 36, 5020 Salzburg, Austria

Correction to: *Scientific Reports* 10.1038/s41598-024-68145-9, published online 1 August 2024

The original version of this Article contained errors, where the values were incorrectly processed.

As a result, in the section “Victimization effects on need for ingroup agency and communion”,

“The results show that structural victimization salience did not affect participants need for ingroup agency or communion, main effect, *F*(1,432) = 55.19, *p* < 0.001, η^2^ = 0.11, within-between interaction.”

now reads:

“The results show that structural victimization salience did not affect participants need for ingroup agency or communion, main effect, *F*(1,432) = 0.35, *p* = 0.554, η^2^ = 0.001. within-between interaction.”

In addition, in the section “Effects of empowerment on reconciliation and cooperation”, the degrees of freedom for the F-tests for ‘reconciliation’ and ‘cooperation’ were incorrectly given as ‘432’. The correct values are 428 for ‘reconciliation’ and 427 for ‘cooperation’.

Also, in section “Exploratory analysis: need for ingroup agency explains increased reconciliation in victims of climate change”, two regression coefficients and their corresponding statistical tests were incorrectly reported in the text.

As a result:

“In line with a needs-based model approach, we found support for the moderated mediation for ingroup agency, as indicated by the significant interaction of empowerment by ingroup agency, b = 0.15, t(432) = 5.82, p < 0.001, but not for ingroup communion, b = − 0.05, t(432) = − 0.38, p = 0.700, or individual need for control, b = − 0.07, t(432) = − 0.62, p = 0.536. Perception of collective victimhood were associated with an enhanced motivation to pursue agentic intergroup goals, b = 0.17, t(432) = 5.82, p < 0.001, which in turn decreased the willingness to reconcile with the older generation when the younger’s need for ingroup agency was not addressed by empowering messages, IE 95% CI [− 0.16, − 0.05].”

Now reads:

“In line with a needs-based model approach, we found support for the moderated mediation for ingroup agency, as indicated by the significant interaction of empowerment by ingroup agency, *b* = 0.60, *t*(432) = 3.41, *p* < 0.001, but not for ingroup communion, b = − 0.05, t(432) = − 0.38, p = 0.700, or individual need for control, b = − 0.07, t(432) = − 0.62, p = 0.536. Perception of collective victimhood were associated with an enhanced motivation to pursue agentic intergroup goals, *b* = 0.15, *t*(432) = 5.39, p < 0.001, which in turn decreased the willingness to reconcile with the older generation when the younger’s need for ingroup agency was not addressed by empowering messages, IE 95% CI [− 0.16, − 0.05].”

The original Article has been corrected.

