# Supplementary material for: Empowering the younger generation increases their willingness for intergenerational reconciliation in the context of climate change
Source: Sci Rep. 2024 Aug 1;14:17825. doi: 10.1038/s41598-024-68145-9 (PMC11294615; doi:10.1038/s41598-024-68145-9)
Supplement: Supplementary file 1 — Supplementary Information. [file 41598_2024_68145_MOESM1_ESM.pdf]

Supplementary Material

1. Principal component analysis on agency and communion intergroup goals

To measure need for agency, we included all four items from the dimensions “be authorative” and “be respected” and added two items from the dimension “be tough” from the Circumplex Scales of Intergroup Goals (Locke et al., 2014). To measure need for communion, we included all four items from the dimensions “be cooperative” and “be conflict-avoidant” and added two items from the dimension “be understanding”. The selection was based on whether item formulations fitted to the context of structural victimization due to climate change. To build our scales for need for agency and need for communion, we conducted a principal component analysis with varimax-rotation on all items. The iteration termined normally and four factors with an Eigenvalue > 1 were extracted. The first factor explained 22% of variance, all items from the high agentic “be authorative” and “be respected” dimensions loaded on that factor, with loadings > .537. Therefore, we termed that factor “need for ingroup agency”. The second factor explained 17% of variance, all items from the high communal “be cooperative” and “be understanding” dimensions loaded on that factor, with loadings > .434. Therefore, we termed that factor “need for ingroup communion”. All items from the “conflict-avoidant” dimension built the third factor, 13% variance explained, and the items from the “be tough” dimension built the fourth factor, 9% variance explained. Based on this factor analysis, we used the first and the second factor to measure need for ingroup agency and need for ingroup communion.

Supplementary Figure 1

Screeplot

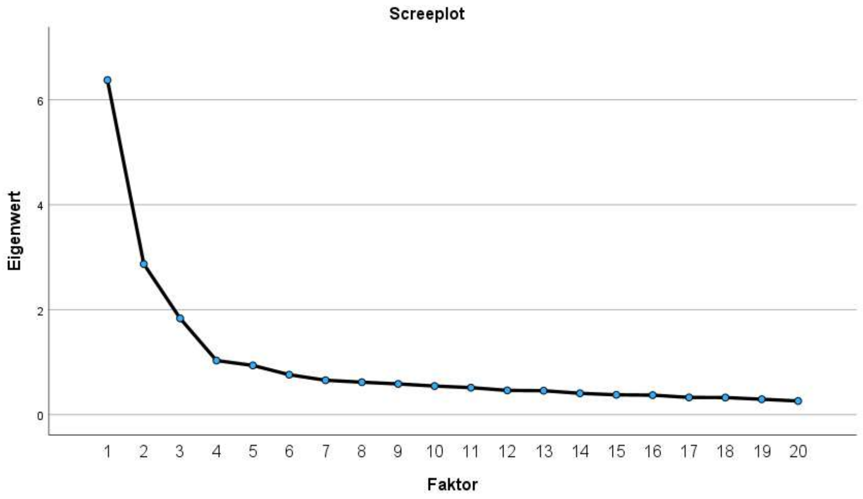

S Table 1

Rotated solution principal component analysis

Items "For us as young generation it is important that…"	Component 1 "Need for Ingroup Agency"	Component 2 "Need for Ingroup Communion"	Component 3 "Conflict avoidance"	Component 4 "Cooperation"
We are assertive	.651	.062	.039	.375
We are decisive	.689	.171	-.040	.276
We are appear confident	.666	.129	-.095	.406
The older generation sees us as capable	.537	.427	.061	.210
The older generation respects what we have to say	.811	.113	.071	-.058
We have the opportunity to express our opinion	.710	.209	-.078	-.118
The older generation listen to what we have to say	.783	.108	.096	-.095
The older generation recognizes our responsibility	.595	.391	.052	.174
we appreciate what the older generation has to offer	.126	.775	.170	.049
We understand their point of view	.176	.791	.088	-.006
we are able to compromise	.311	.601	.339	-.214
We show concern for their welfare	.124	.733	-.046	.218
We are cooperative	.385	.596	.293	-.186
They feel that we are all on the same team	.392	.434	.155	.050
We avoid conflict	-.039	.104	.815	.148
The older generation get not angry with us	-.119	.347	.588	.440
We not make them angry	-.176	.356	.545	.468
We no get into arguments	.138	.133	.822	.007
We are tough	.215	.005	.159	.718
We not appear vulnerable	.152	-.072	.441	.529

Note. Principal Component Analysis with varimax-rotation of all items. The iteration termined normally and four factors with an Eigenvalue > 1 were extracted.

2. Answers to open question "Opinion on Intergenerational Conflict"

S Table 2

Opinion about the existence of a generation conflict in the context of climate change

Conflict		No conflict		Other	
Unspecific conflict	221	No conflict	52	unspecific	41
Structural inequality	26	Individual responsibility	18	No answer	23
Dual conflict	44			Never thought about that	9
	291		70		73
	67.1%		16.1%		16.8%

Note. Responses of *N* = 434 participants were coded. A table with all open answers in original wording is available on OSF. Link is provided in the main manuscript.
